# Association of Metabolic Diseases and Moderate Fat Intake with Myocardial Infarction Risk

**DOI:** 10.3390/nu16244273

**Published:** 2024-12-11

**Authors:** Junyu Zhou, Meiling Liu, Sunmin Park

**Affiliations:** 1 Institute of Advanced Clinical Medicine, Peking University, Beijing 100191, China; zjy888zjy888@gmail.com; 2 Department of Chemical Engineering, Shanxi Institute of Science and Technology, Jincheng 048011, China; liumeiling@sxist.edu.cn; 3 Department of Food and Nutrition, Obesity/Diabetes Research Center, Hoseo University, 20 Hoseoro97bungil, BaeBang-Yup, Asan 31499, Republic of Korea

**Keywords:** myocardial infarction, polygenic risk score, metabolic syndrome, single-cell RNA sequencing, precision medicine, fat

## Abstract

Background: Myocardial infarction (MI) can range from mild to severe cardiovascular events and typically develops through complex interactions between genetic and lifestyle factors. Objectives: We aimed to understand the genetic predisposition associated with MI through genetic correlation, colocalization analysis, and cells’ gene expression values to develop more effective prevention and treatment strategies to reduce its burden. Methods: A polygenic risk score (PRS) was employed to estimate the genetic risk for MI and to analyze the dietary interactions with PRS that affect MI risk in adults over 45 years (*n* = 58,701). Genetic correlation (rg) between MI and metabolic syndrome-related traits was estimated with linkage disequilibrium score regression. Single-cell RNA sequencing (scRNA-seq) analysis was performed to investigate cellular heterogeneity in MI-associated genes. Results: Ten significant genetic variants associated with MI risk were related to cardiac, immune, and brain functions. A high PRS was associated with a threefold increase in MI risk (OR: 3.074, 95% CI: 2.354–4.014, *p* < 0.001). This increased the risk of MI plus obesity, hyperglycemia, dyslipidemia, and hypertension by about twofold after adjusting for MI-related covariates (*p* < 0.001). The PRS interacted with moderate fat intake (>15 energy percent), alcohol consumption (<30 g/day), and non-smoking, reducing MI risk in participants with a high PRS. MI was negatively correlated with the consumption of olive oil, sesame oil, and perilla oil used for cooking (rg = −0.364). MI risk was associated with storkhead box 1 (*STOX1*) and vacuolar protein sorting-associated protein 26A (*VPS26A*) in atrial and ventricular cardiomyocytes and fibroblasts. Conclusions: This study identified novel genetic variants and gene expression patterns associated with MI risk, influenced by their interaction with fat and alcohol intake, and smoking status. Our findings provide insights for developing personalized prevention and treatment strategies targeting this complex clinical presentation of MI.

## 1. Background

Myocardial infarction (MI) is as a multifaceted pathological condition driven by a complex and dynamic interplay of genetic predispositions, environmental exposures, chronic inflammatory processes, metabolic dysregulation, and lifestyle-associated risk factors. Recent genomic and epidemiological studies have revealed MI as a sophisticated molecular disease beyond traditional risk factor models [1,2]. East Asian populations present a unique MI epidemiological profile distinct from Western countries, characterized by lower overall incidence rates but increasingly alarming trends [3,4]. Specifically, East Asian populations demonstrate several notable characteristics: MI onset at younger ages, a more pronounced genetic component, and distinctly different metabolic risk factor distributions compared to Western populations [5,6]. These differences are driven by rapid urbanization, significant dietary transitions, and evolving lifestyle patterns in countries like China, South Korea, and Japan [7]. The complex interplay of genetic and environmental factors in East Asian populations offers unprecedented opportunities to enhance MI risk prediction, prevention, and personalized treatment strategies.

The intricate relationship between lifestyle factors and genetic susceptibility to MI represents a critical area of scientific investigation. Emerging research demonstrates that adherence to healthy lifestyle habits can significantly mitigate MI risk, particularly in individuals with genetic predispositions [8]. Tailored lifestyle interventions have shown promising potential in reducing MI risk, especially among high-risk populations. Integrating genetic markers with lifestyle interventions offers a more comprehensive approach to MI prevention and management [9,10].

The complex relationship between myocardial infarction (MI), metabolic diseases, and lifestyle factors reveals multiple influences on cardiovascular health. Extensive epidemiological studies, such as the Wisconsin Longitudinal Study, have consistently identified critical risk factors for MI, including hypertension, dyslipidemia, type 2 diabetes (T2DM), and obesity [11]. An Australian study further highlighted how the duration of T2DM can predict cardiovascular events and mortality, especially in older populations [12]. These findings emphasize the need for a comprehensive approach integrating genetic and lifestyle factors in MI prevention. Our study uniquely investigates the genetic correlations between MI and multiple metabolic conditions, focusing on the interactions of three or more chronic metabolic conditions (3GO).

Genetic research has identified specific variants associated with MI, such as rs11614913 in the microRNA 196a-2 (MIR196A2) gene, which has been linked to increased MI risk in European populations [13]. This variant potentially influences MI pathology by regulating genes involved in atherosclerosis and coronary artery disease [13,14]. Colocalization analysis has further illuminated the complex interactions between genetic variants and regulatory elements in MI development.

Cutting-edge single-cell RNA sequencing (scRNA-seq) techniques have enabled researchers to map gene expression profiles related to MI [15], providing unprecedented insights into cell-specific genetic factors. Our primary objective was to comprehensively examine the genetic determinants of MI and associated comorbidities in East Asian populations. By employing genetic correlation, colocalization analysis, and cell-type-specific expression pattern investigations, we sought to uncover the intricate genetic architecture of MI. Ultimately, our research aims to contribute to more precise and efficient preventive and therapeutic approaches tailored specifically to East Asian populations.

## 2. Methods

### 2.1. Data Sources and Preprocessing

This study utilized MI data from the Korean Genome and Epidemiology Study (KoGES) [16], a population-based cohort study conducted by the Korea National Institute of Health (KNIH) and the Korea Centers for Disease Control and Prevention (KCDC). The KoGES included 58,701 adults recruited between 2004 and 2013. The study protocol was approved by the institutional review board (IRB) of the KNIH (KBP-2015-055), and participants provided written informed consent. Additional approval was obtained from the IRB of Hoseo University (1041231-150811-HR-034-01). Information on the participants’ characteristics, lifestyle, and medications was collected through a questionnaire [16], providing comprehensive genotypic data of MI patients and control subjects.

The KoGES study collected sociodemographic characteristics, anthropometric measurements, and biochemical data. The sociodemographic questionnaire captured the participants’ residence area, gender, and age, as well as smoking, drinking, and exercise habits. Physical activity was assessed based on participants engaging in moderate-intensity physical activity for at least 150 min per week [17,18]. Smoking status was categorized as current, former, or non-smoking, determined by whether participants had smoked more than 20 cigarettes over their lifetime, with former smokers defined as those who had previously smoked but had not done so in the last 6 months [19]. Alcohol consumption was calculated by multiplying the reported amount and type, such as beer, soju, rice wine, wine, or whisky, consumed during each alcohol-drinking event by the frequency of those events [20]. Body mass index (BMI) was calculated by body weight (kg)/height (m)^2^ [21]. Blood pressure was measured using a standardized protocol, and fasting blood samples from the participants after no food for at least 12 h were collected for biochemical analysis, including the measurement of glucose and HbA1C, total cholesterol, high-density lipoprotein cholesterol (HDL), and triglycerides using an automatic analyzer [19]. Serum LDL concentration was calculated using the Freidman equation, which remains the most widely used method for estimating LDL concentration levels in clinical practice [22]. When serum triglyceride concentration was greater than 500 mg/dL, serum LDL concentration was not calculated. The characteristics and health indicators of the participants were measured while adhering to established standard methods to ensure reliability and validity.

### 2.2. Definition of MI and Comorbid Diseases

Participants were stratified into an MI group based on the presence of a documented, hospital-confirmed diagnosis of MI within their medical history, comprising a total of 1998 subjects. MI diagnosis was confirmed through comprehensive medical documentation, requiring a definitive physician diagnosis, while those without MI and three or more comorbidities (3GO) constituted the non-MI group (*n* = 26,032). Further, subgroups were established based on specific comorbidities (3GO). The MI + HT group comprised individuals with a history of MI and hypertension, with hypertension determined according to the criteria outlined by the International Society of Hypertension (systolic blood pressure [SBP] ≥ 140 mmHg and diastolic blood pressure [DBP] ≥ 90 mmHg) and taking hypertension medication [23]. The MI + T2DM group comprised individuals with a history of MI, fasting blood glucose levels ≥ 126 mg/dL, and taking anti-diabetic agents [24]. Individuals in the MI + DL group included those with a history of MI and dyslipidemia, characterized by serum triglycerides, total cholesterol, or HDL concentrations ≥ 200 mg/dL, ≥ 250 mg/dL, or <40 mg/dL, respectively, and taking hypolipidemic agents as per the guidelines of the International Society of Endocrinology [19]. The MI + OB group comprised individuals with a history of MI and obesity, the latter defined by a BMI ≥ 25.0 kg/m^2^ [17]. Lastly, the 3GO category encompassed individuals with three or more of the following conditions: hypertension, T2DM, dyslipidemia, and obesity. Notably, 3GO represents a severe state of metabolic syndrome (MetS).

### 2.3. Food and Nutrient Intake Assessments

The Semi-Quantitative Food Frequency Questionnaire (SQFFQ), containing 106 everyday Korean food items, was employed and validated for the KoGES. Participants reported their food intake frequency and portion sizes over the past year to a skilled technician. Intake for each item was calculated by multiplying the consumption frequency by portion size, with the daily intake being the sum of all 106 items. Nutrient intake was analyzed using the CAN-Pro 3.0 program developed by the Korean Nutrition Society [11] and compared with dietary reference intake standards.

### 2.4. Genotyping and Quality Control: Genomic Analysis and Quality Assurance Procedures

Genomic DNA was extracted from peripheral blood monocytes following standard protocols. Single-nucleotide polymorphisms (SNPs) were determined using the Korean Chip (K-CHIP) [25], which is tailored for the Korean population and contains 830,000 SNPs (Affymetrix, Santa Clara, CA, USA). The K-CHIP was developed with stringent quality control by the Center for Genome Science at KNIH to ensure data reliability. Genotyping accuracy was assessed using Bayesian Robust Linear Modeling with the Mahalanobis (BRLMM) Distance algorithm, with rigorous quality control measures that excluded low-quality SNPs. The SNP exclusion criteria included genotyping accuracy < 98%, missing genotype calls (≥4%), excessive heterozygosity (>30%), an unsatisfied Hardy–Weinberg equilibrium (HWE; *p* <  0.05), and minor allele frequency (MAF) below 1%. Only high-quality SNPs meeting the criteria were retained for subsequent analyses, ensuring robust and meaningful results [26].

The SNP-related genes were identified using the SNP and copy number variant (CNV) Annotation Database (SCAN) site (http://scandb.org/newinterface/, accessed on 24 April 2024), with linkage disequilibrium (LD) confirmation using LocusZoom [27] (r^2^ < 0.3). We extensively explored biological datasets and utilized GeneMANIA (http://genemania.org/, accessed on 16 May 2024) for gene identification, including protein–protein, protein–DNA, genetic interaction, pathway, response, and gene and protein expression data.

### 2.5. PRS Generation via the GMDR Program

From the carefully selected SNPs associated with MI onset, we utilized the generalized multifactor dimensionality reduction (GMDR) program available at http://ibi.zju.edu.cn/software to generate a PRS predicting genetic predisposition to MI [28]. The GMDR method offers a unique advantage in handling complex gene–gene interactions, providing a more comprehensive genetic risk assessment than traditional single-gene association analyses. The PRS represents the cumulative impact of multiple SNPs on MI risk. Based on meeting the criteria for training balance accuracy (TRBA), test balance accuracy (TEBA), and cross-validation consistency, the optimal model guided our selection of the SNPs associated with MI to construct the PRS. Genetic variants in the chosen model exhibited a strong association of gene–gene interactions with MI risk. The PRS calculation involved summing the risk alleles from the SNPs in the best model, with minor alleles counted as 1 for SNPs with an odds ratio (OR) > 1, indicating increased MI risk. Conversely, major alleles were counted as 1 for SNPs with an OR between 0 and 1.

We encoded selected genotype data for GMDR analysis to identify the synergistic genetic variant combinations linked to MI. The PRS calculation considers the complex nature of genetic variants, which are composed of non-risk, heterozygote, and risk alleles. Our PRS construction method carefully accounts for the genetic variant composition, categorizing participants based on their allele configurations. Each genetic variant was evaluated considering non-risk, heterozygote, and risk alleles, with the categorization into low PRS (2–7), medium PRS (8–10), and high PRS (11–15) directly corresponding to genetic variant allele combinations. This approach allows a more precise translation of genetic risk into clinically meaningful categories. By stratifying the PRS based on the underlying genetic variant allele types, we can more accurately reflect the nuanced genetic risk profiles, provide a robust method for risk stratification, and improve the potential for personalized risk assessment. The PRS calculation involved summing the weighted number of risk alleles for each variant according to its encoded genotype in the predefined gene combination. This method transforms complex genetic data into a structured, interpretable format that can be more directly applied to understanding individual MI risk.

### 2.6. Descriptive Statistical Analysis and Lifestyle Interaction Analysis

We utilized Stata Statistical Software (Release 17: StataCorp LLC, College Station, TX, USA) to examine categorical variable frequency distributions and conducted Chi-square tests for variables such as gender, smoking status, and alcohol consumption. Frequencies and percentages were calculated for each categorical variable. Descriptive statistical analysis, using Stata, assessed continuous variables like age, physical activity level, and income. After adjustments for covariates, including age, gender, residence area, BMI, daily energy intake, physical activity, smoking status, and alcohol consumption, we computed the mean and standard deviation (SD). Significance was determined at *p* < 0.05, with values below this indicating statistical significance. We employed Chi-square tests for categorical variable frequency distribution analysis. The continuous variables were assessed through a one-way analysis of variance (ANOVA), with covariate adjustments for age, gender, residence area, and BMI to identify the differences between the control and MI groups. Significant differences among the PRS categories were assessed using one-way ANOVA with the same adjustments. Multiple comparisons among the groups were conducted using Duncan’s multiple comparison test. GWASs were used to find the SNPs associated with MI risk, and Bonferroni correction was applied to the level of statistical significance to find significant SNPs. Logistic regression in Stata explored PRS-MI associations, with PRS as an independent variable, MI onset status as the dependent variable, and covariates including residential area, age, gender, physical activity, education, income, smoking, and alcohol consumption. The adjusted OR and 95% confidence intervals (CIs) were calculated for the medium-PRS and high-PRS groups, with the low-PRS group as the reference. To ensure the statistical reliability of our research, we conducted a rigorous sample size calculation. The calculation process detected the parameters with the minimum genetic effect, as well as variations in genotype and allele frequency. Considering the heterogeneity of the population’s genetic background, minimum sample requirements were assessed to detect statistically significant associations.

We investigated the interaction between lifestyle factors (nutrient intake, physical activity, smoking, alcohol consumption) and PRS in MI risk using logistic regression models, including covariates such as residential area, age, gender, BMI, smoking, alcohol consumption, coffee consumption, energy intake, fat and carbohydrate intake, and physical activity to mitigate confounding effects. Lifestyle factors were categorized into low and high groups based on designated levels. The smoking category was divided into current and former smokers and non-smokers. Daily energy, protein, fat, carbohydrate, calcium, sodium, and coffee consumption were also used as interaction factors. Statistical significance was set at *p* ≤ 0.05.

### 2.7. Estimating Genetic Correlation Between MI and Other Phenotypes in East Asian Populations Using LDSC Software

Using the GWAS datasets available in the public domain, we utilized the linkage disequilibrium score regression (LDSC v1.0.1) software, downloaded from https://github.com/bulik/ldsc, to estimate the genetic correlation between MI and various phenotypes [29]. The LDSC analysis provided systematic insights into cross-phenotype genetic associations, helping to uncover potential shared genetic mechanisms. These datasets include reference SNP cluster ID (rs) number, chromosome number, position, effect allele, null allele, and the OR, along with the *p*-value of the HWE for both MI and other traits. Japanese population GWAS data were sourced from the BioBank Japan (BBJ) Project [30] (http://jenger.riken.jp/result), and Korean population data were obtained from KoGES [16] (https://koges.leelabsg.org/). We utilized East Asian ancestry population data from the 1000 Genomes East Asian dataset as a reference [31]. Precomputed LD scores and weights for East Asian populations were obtained from the 1000 Genomes Project Phase 3 data (https://console.cloud.google.com/storage/browser/broad-alkesgroup-public-requester-pays). The data underwent quality control procedures, including ensuring MAF > 0.05 in HapMap 3 and removing low-imputation-quality SNPs and non-matching alleles [32]. SNPs with χ^2^ > 80 were excluded to minimize the outlier effect in LD score regression [33]. The traits data were categorized into health indicators, diet, and lifestyle factors. The genetic correlation, representing the extent of shared genetic influence between two phenotypes, ranged from −1 to 1 [34].

### 2.8. Colocalization Analysis Based on Bayesian Inference

The colocalization employed a Bayesian framework, incorporating data from GWASs and expression quantitative trait loci (eQTL) mapping to assess the consistency of the shared causal variation among two or more independently associated signals within a region [35]. This approach enabled us to identify potential gene regulatory mechanisms crucial in MI pathogenesis. Our research approach aimed to identify SNPs significantly associated with MI from GWASs while ensuring that their linkage equilibrium did not exceed a predetermined threshold (r^2^ < 0.2). Subsequently, colocalization analysis was conducted for each SNP, integrating both GWAS and eQTL signals and assessing their overlap within a 1-megabase (mb) range upstream and downstream of the gene. We leveraged the eQTL data from the Genotype-Tissue Expression (GTEx) project v6−v8 datasets, along with additional datasets from 37 sources, comprising a total of 31,684 individuals (http://www.eqtlgen.org/eqts.html). Using MAGMA v1.10, we identified the SNPs significantly associated with MI [36]. Trait selection for analysis was based on genotype–phenotype associations obtained from the PhenoScanner database (https://github.com/phenoscanner/phenoscanner). The Coloc package in R language computed posterior probabilities and approximate Bayes factors for each SNP [37], providing insights into evidence of colocalization (https://chr1swallace.github.io/coloc). Colocalization posterior probability was determined based on SNP statistics, such as the *p* value, MAF, and sample size. Finally, regional association maps were generated using LocusZoom.js (https://github.com/statgen/locuszoom), incorporating linkage disequilibrium information from the GTEx data [38]. The code for the plots of colocalization is available at the provided GitHub repository (https://github.com/Benjamin-JHou/postGWAS_Colocalization).

### 2.9. MI-Related Gene Expression and Scoring in Single-Cell RNA-Seq Datasets

The scRNA-seq data from myocardial tissue samples were analyzed to explore cell-type-specific gene expression profiles related to MI [39]; the data can be downloaded at https://www.heartcellatlas.org/#publication. Raw data in H5AD format, containing expression matrices and metadata, were processed using scanpy in Python 3.11. Additionally, a z-score file for MI-associated genes was integrated to compute a custom disease relevance score for each cell. This score, reflecting each cell’s association with the MI phenotype, was calculated as the weighted sum of expression levels for genes common to both the scRNA-seq data and the MI gene set. Weights were derived from the MI scores, indicating each gene’s contribution to the disease phenotype. The disease relevance score for each cell was computed using a weighted sum of the expression levels of the MI-associated genes. Specifically, for a cell *c* and a set of common genes *G*, the disease relevance score *S_c_* is calculated as follows:

$$S_{c}=\sum_{g\in G}E_{c,g}\cdot W_{g}$$

where *E_c,g_* represents the expression level of gene *g* in cell *c*, and *W_g_* is the MI score of gene *g*, reflecting its contribution to the MI phenotype. The relevance scores were normalized to a 0–10 scale to facilitate comparison across cells using the following formula:

$$S_{c,norm}=\frac{S_{c}-\min(S)}{\max(S)-\min(S)}\times 10$$

The specific methods and steps are explained in detail in GZSper (GWAS Z-score to Single-cell Phenotypes for Expression Research). The code for the analysis and plots is available at the provided GitHub repository (https://github.com/Benjamin-JHou/GZSper).

Dimensionality reduction was performed using Principal Component Analysis (PCA), followed by Uniform Manifold Approximation and Projection (UMAP) to visualize cellular heterogeneity. These steps allowed for examining disease relevance across various cell types, providing insights into the molecular mechanisms underlying MI.

## 3. Results

### 3.1. Anthropometric and Biochemical Characteristics of MI and Its Comorbidities

As seen in Supplementary Table S1, compared to the control group, the patients in the MI group had higher waist circumferences by 4.2 cm, body fat by 2.8%, fasting blood glucose by 6.1 mg/dL, HbA1c by 0.45%, and SBP by 3 mmHg (healthy participants). The MI plus comorbidity groups also showed worse metabolic dysfunctions than the MI group. The average age of the patients in the MI + T2DM subgroup was higher than the other subgroups at 61.3 years (Supplementary Table S1). Discrepancies in gender distribution were observed across the control, MI, and MI plus comorbidity subgroups, with women predominantly represented in the control group, whereas the prevalence of men was higher in the MI plus comorbidity subgroups (Supplementary Table S1). Among all the subgroups, patients in the MI + T2DM group were the least active, with the lowest frequency of performing physical exercise, while those in the MI + 3GO group showed the highest frequencies of coffee and alcohol intake (Supplementary Table S2). Conversely, smoking frequency was lowest in the control group, but fat intake as part of total daily intake was the highest in this group (Supplementary Table S2).

### 3.2. MI-Risk Genes from GWASs and GMDR

**Figure 1:**
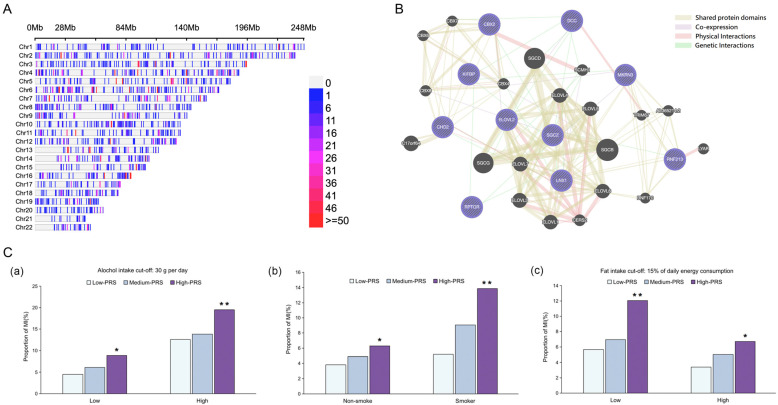
SNP-SNP interactions and PRS and lifestyle interactions. (**A**) Distribution of SNPs within a 1 Mb window size across chromosomes 1 to 22. SNPs were filtered based on a significance threshold of *p*-value < 0.0001. Adjacent regions with high SNP density (≥50 SNPs) are highlighted in red, while locations with fewer SNPs are displayed in a gradient from red to blue, indicating decreasing density. SNPs that did not pass the filtering criteria are shown in gray, serving as a reference for genomic regions without significant associations. (**B**) Interaction of genes: a comprehensive interaction diagram of ten key genetic elements. (**C**) Frequencies of MI in participants with low, medium, and high polygenic risk score (PRS) based on the optimal 9 risk alleles: *LNX1*_rs2616417, *ELOVL2*_rs75105616, *SGCZ*_rs73201298, *KIFBP*_rs3864814, *MKRN3*_rs56730421, *CHD2*_rs201915192, *RNF213*_rs1410411669, *RPTOR*_rs7224758, and *DDC*_rs77235945. (**a**) Participants categorized by alcohol intake (cut-off: 30 g/d). (**b**) Participants categorized by smoking status (non-smokers versus current and former smokers). (**c**) Participants categorized by fat intake (cut-off: 15% of daily energy consumption). Significant differences between low- and high-PRS groups: * *p* < 0.01, ** *p* < 0.001. MI: myocardial infarction; *LNX1*: ligand of numb-protein X 1; *ELOVL2*: ELOVL fatty acid elongase 2; *SGCZ*: sarcoglycan zeta; *KIFBP*: KIF-binding protein; *MKRN3*: makorin ring finger protein 3; *CHD2*: chromodomain-helicase-DNA-binding protein; *CBX2*: chromobox protein homolog 2; *RNF213*: ring finger protein 213; *RPTOR*: regulatory-associated protein of mTOR; *DDC*: dopa decarboxylase.

To investigate the genomic distribution of significant SNPs, we analyzed their density across chromosomes 1 to 22 within a 1 Mb window size (Figure 1A). After applying a significance threshold of *p*-value < 0.0001, the SNPs were visualized by density. Regions with high SNP density (≥50 SNPs) are prominently marked in red, indicating potential hotspots of genetic association. In contrast, regions with fewer significant SNPs are marked by a gradient from red to blue, with blue representing lower SNP densities. Non-significant SNPs that did not meet the *p*-value threshold are plotted in gray, revealing background genomic variation. This distribution highlights key loci for further investigation and suggests regions with potential biological significance. We identified 10 significant SNPs that met the SNP quality and SNP-SNP interaction criteria. These SNPs, detailed in Supplementary Table S3, included ligand of numb-protein X 1 (*LNX1*), ELOVL fatty acid elongase 2 (*ELOVL2*), sarcoglycan zeta (*SGCZ)*, KIF-binding protein *(KIFBP*), makorin ring finger protein 3 (*MKRN3*), chromodomain-helicase-DNA-binding protein (*CHD2*), chromobox protein homolog 2 (*CBX2*), ring finger protein 213 (*RNF213*), regulatory-associated protein of mTOR (*RPTOR*), and dopa decarboxylase (*DDC*). Figure 1B illustrates the interaction among these ten genes, demonstrating shared protein domains, genetic interactions, co-expression, and physical interactions. Using the GMDR method, the genetic variants of the SNPs of the 10 genes with intricate genetic interactions were identified. Model 9, including nine genetic variants, presented in Supplementary Table S4, satisfied the criteria for GMDR, with an adjusted test equilibrium accuracy of 10 (*p* = 0.0010). This result explains the significant and potentially synergistic roles of these nine genetic variants in influencing MI risk.

### 3.3. Correlations with Physiological Characteristics in Participants with High PRS

Table 1 presents the metabolic characteristics of the participants in the three PRS groups across the control and MI groups. Compared to the low-PRS group, individuals with a high PRS demonstrated higher BMI, waist circumference, hip circumference, blood HbA1c, fasting blood glucose, total cholesterol, triglycerides, SBP, and DBP only in the MI group (not in the control group). Conversely, in participants with MI, the high-PRS group exhibited lower serum HDL cholesterol levels than the low-PRS group.

### 3.4. Logistic Regression Analysis of PRS and MI with Comorbid Diseases

There was a positive significant association of high PRS with MI risk (OR: 3.074, 95% CI: 2.354–4.014, *p* < 0.001) in the logistic regression, adjusting for confounding variables such as age, sex, residence area, BMI, energy intake, alcohol and coffee consumption, and physical activity (Table 2). Additionally, MI + HT (OR: 2.045, 95% CI: 1.747–2.429, *p* < 0.001), MI + T2DM (OR: 2.428, 95% CI: 1.675–3.492, *p* < 0.001), MI + DL (OR: 1.975, 95% CI: 1.499–2.447, *p* < 0.001), MI + OB (OR: 2.238, 95% CI: 1.648–2.776, *p* < 0.001) and 3GO (OR: 1.915, 95% CI: 1.132–2.019, *p* < 0.01) also showed a positive association with high PRS.

### 3.5. Interaction Between PRS and Lifestyle Factors Influencing MI Risk

Table 3 details the interaction between the PRS and key lifestyle factors after adjusting for MI and relevant covariates. Dietary fat intake, alcohol, and smoking revealed complex genetic (PRS)–lifestyle interactions that are crucial for understanding MI pathogenesis and risk accumulation. Figure 1C illustrates the distribution of low-, medium-, and high-PRS participants across the MI and MI plus comorbidity groups. High-PRS participants were at an increased risk of MI with low fat intake, high alcohol intake, and smoking.

### 3.6. Genetic Correlations of MI with Health Indicators, Diet, and Lifestyle Factors

**Figure 2:**
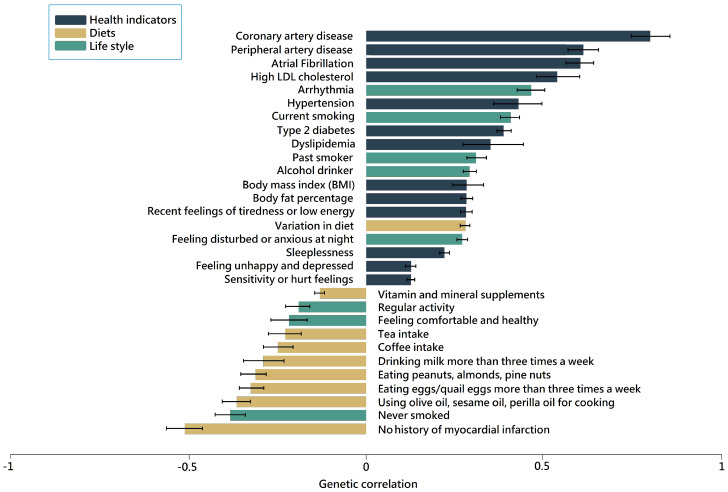
Genetic correlations in Asian ancestry between MI (*n* = 28,030) and other traits after Bonferroni correction. The traits associated with MI are divided into three categories: dark blue, brown, and green color bars indicate health indicators, dietary factors, and lifestyle factors, respectively. MI: myocardial infarction.

The genetic correlation coefficients between MI and other traits, including MI-related diseases, diets, and lifestyles, are shown in Figure 2, and their false discovery rates (FDR) are available in Supplementary Table S5. Significant associations between MI and various traits that met r_g_ > 0.4 or <−0.2 (FDR < 5%) were found for coronary artery disease (r_g_ = 0.76, *p* = 3.55 × 10^−130^), peripheral artery disease (r_g_ = 0.61, *p* = 1.15 × 10^−32^), atrial fibrillation (r_g_ = 0.61, *p* = 1. 52 × 10^−51^), hyper-LDL cholesterolemia (r_g_ = 0.54, *p* = 8.47 × 10^−28^), arrhythmia (r_g_ = 0.46, *p* = 4.34 × 10^−09^), hypertension (r_g_ = 0.43, *p* = 1.72 × 10^−30^), and current smoking status (r_g_ = 0.41, *p* = 1.59 × 10^−06^). These associations indicate the complex genetic mechanisms underlying MI. Conversely, certain dietary habits, including the use of olive oil, sesame oil, and perilla oil for cooking (r_g_ = −0.36, *p* = 1.78 × 10^−7^), the frequent consumption of eggs/quail eggs (r_g_ = −0.32, *p* = 3.53 × 10^−7^), peanuts, almonds, pine nuts (r_g_ = −0.31, *p* = 1.39 × 10^−7^), and milk (r_g_ = −0.30, *p* = 5.09 × 10^−7^), as well as coffee (r_g_ = −0.25, *p* = 2.95 × 10^−5^) and tea intake (r_g_= −0.23, *p* = 0.002), had negative associations with MI risk. This suggests that specific ingredients in our diet could be potential protective modulators of MI.

### 3.7. Regulatory Mechanisms of MI-Associated Genetic Variants Through Colocalization Analysis

**Figure 3:**
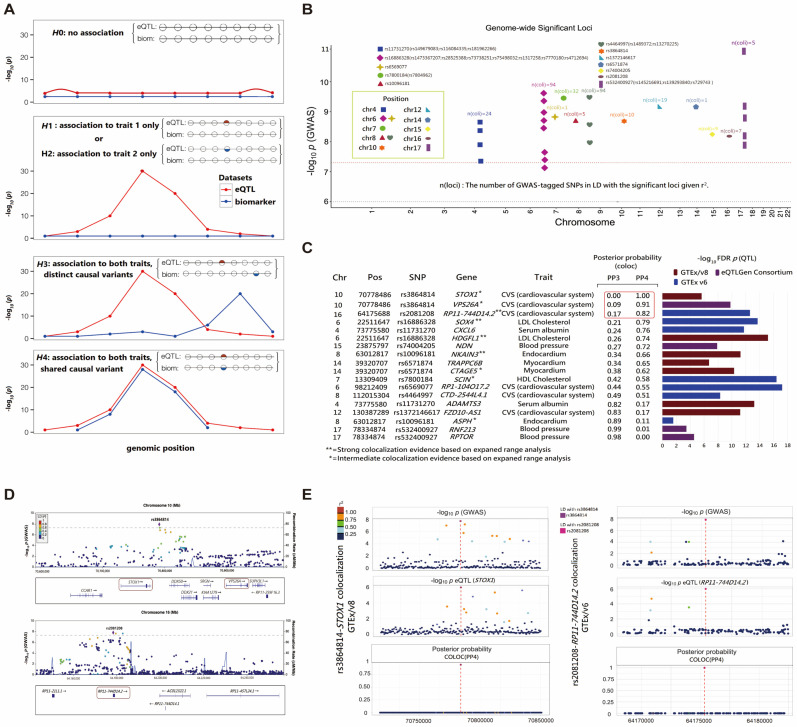
Colocalization analysis of SNPs associated with MI in eQTL datasets. (**A**) Schematic diagram of colocalization analysis under different hypotheses (H0, H1/H2, H3, H4). A binary vector representing the number of shared variants in each feature’s region. The value on the *y*-axis indicates whether the variation has a causal relationship with the disease. Matching positions of eQTL (red) and biomarkers (blue) indicate the same causal SNP, while different positions indicate that the causal SNP of the dataset is different. (**B**) Combined Manhattan plot showing 12 mapped distinct loci and 28 genome-wide significantly associated SNPs (*p* < 5 × 10^−8^), as well as the number of suggestive SNPs identified in each genome. (**C**) Data supporting a single variant (PP4 > 80%) affecting both traits are identified by a red border. High association evidence genes (eQTL ± 1MB) from extended range analysis are marked as moderate (*) or strong (**). −Log _10_ FDR p plots of eQTL representing the tissue expression of significantly associated SNPs with corresponding posterior probabilities for GTEx. (**D**) LocusZoom plots mapping the genomic locations of significantly associated SNPs (rs3864814, rs2081208) on chromosome 10 and chr16, providing reliable evidence supporting a colocalization signal on *STOX1-VPS26A* and *RP11-744D4.2.* (**E**) eQTL association plots of colocalization of rs3864814, rs2081208 with *STOX1, RP11-744D4.2* in the corresponding GTEx dataset. GWAS −log _10_ *p* for SNPs corresponding to GTEx, eQTL −log _10_ *p* for *STOX1* and *RP11-744D4.2*, and evidence of causal variation in posterior probabilities are shown, respectively. SNPs: single-nucleotide polymorphisms; MI: myocardial infarction; eQTL: expression quantitative trait loci; PP4: posterior probability 4; FDR: false discovery rates; *STOX1-VPS26A:* storkhead box 1_vacuolar protein sorting-associated protein 26A; GTex: Genotype-Tissue Expression.

In the colocalization analysis, to understand the regulatory mechanisms of MI-associated variants, the eQTL of the genetic variants was divided into four different categories, namely, no association between the eQTL of the genetic variant and the trait (H0), association with trait 1 only (H1) or trait 2 only (H2), association with both traits with distinct causal variants (H3), and association with both traits with shared common causal variants (H4) (Figure 3A). A low *p*-value of the eQTL value and trait indicated a significant association. We identified 12 distinct loci and 28 genome-wide significantly associated SNPs (*p* < 5 × 10^−8^), as shown in Figure 3B. The selected SNPs and associated signals are depicted in Figure 3C. Among them, the posterior probability 3 (PP3) values for rs3864814_storkhead box 1 (*STOX1*), rs3864814_vacuolar protein sorting-associated protein 26A (*VPS26A*), and rs2081208_*RP11-744D14.2* are relatively low at 0.00, 0.09, and 0.17, respectively, indicating a lower probability of potentially different causal variants. In contrast, the PP4 values, all greater than 0.80 (specifically, 1.00, 0.91, and 0.82, respectively), suggest a high probability of association with both traits with shared causal variants. This robust colocalization implies that the same genetic variants are associated with both traits. The genetic variants have a causal relationship with MI. Functionally annotated SNPs (rs3864814 and rs208120) were assigned to genes based on genomic location and chromatin interactions (Figure 3D). GWAS SNP −log_10_ *p*-values corresponding to GTEx, eQTL −log_10_ *p*-values for rs3864814_*STOX1* and rs2081208_*RP11-744D14.2*, and evidence of causal variation in the posterior probabilities were aligned (Figure 3E). Our analysis highlighted an independent association signal at rs3864814, colocalizing with two genes: *STOX1* and *VPS26A*. rs3864814 shares a common causal variant with these genes.

### 3.8. MI-Related Gene Score and Expression of Top Genes Across Different Cell Types

**Figure 4:**
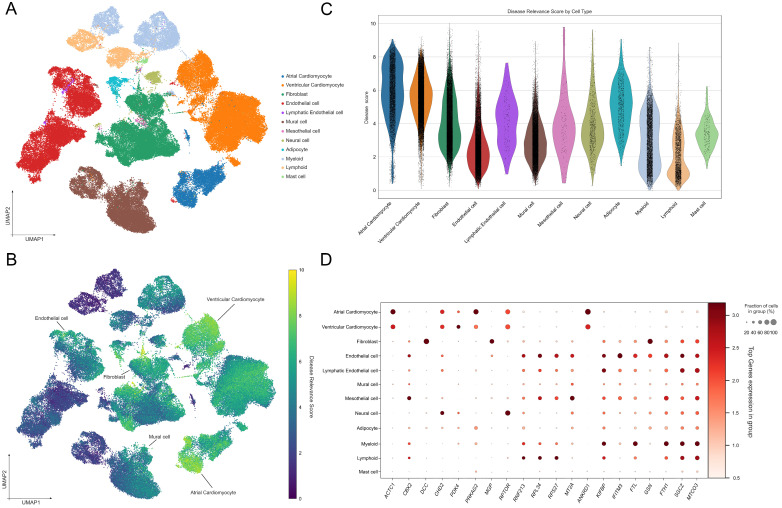
UMAP and gene expression analysis of cardiac cell types and disease relevance scores for MI. (**A**) UMAP visualization of major cardiac cell types; UMAP plot showing 12 major cardiac cell types identified from the Heart Cell Atlas v2 dataset. Each color represents a different cell type, including cardiomyocytes, fibroblasts, endothelial cells, mural cells, and others. (**B**) UMAP of disease relevance scores across cardiac cell types. Cells with higher scores are marked by warmer colors, indicating a stronger association with disease relevance. (**C**) Violin plot of disease relevance scores by cell type. The width of each violin corresponds to the frequency of cells with scores in each cell type. (**D**) Dot plot of key gene expression across cardiac cell types. The size of each dot represents the expression level, while the color intensity indicates the relative gene expression in each cell type.

We applied our gene expression analysis pipeline to the Heart Cell Atlas v2 scRNA-seq dataset, which contains 704,296 cells across various regions of the heart, including the left and right ventricular free walls, left and right atria, apex, septum, sinoatrial node, and atrioventricular node. Using advanced machine learning techniques for clustering and visualization, we identified 12 distinct major cardiac cell types in the UMAP plot (Figure 4A).

In the disease relevance UMAP, atrial and ventricular cardiomyocytes, fibroblasts, endothelial cells, and mural cells exhibited notably high disease relevance scores, indicating their significant involvement in myocardial infarction pathology (Figure 4B). Among the cell types, ventricular cardiomyocytes were the most frequent cell types associated with higher disease relevance scores, as seen in the violin plot (Figure 4C). Interestingly, atrial cardiomyocytes displayed the highest average disease relevance score, suggesting a strong link to the MI phenotype in this cell type.

The expression patterns of key genes across different cell types were visualized using dot plots. Notably, *CHD2* and *RPTOR* showed the highest expression levels in neural cells, *DCC* in fibroblasts, *CBX2* in mesothelial cells, *RNF213* in lymphoid cells, and *KIFBP* in myeloid cells (Figure 4D). These gene-specific expression profiles provide further insights into the molecular mechanisms operating within these diverse cell populations in the context of MI.

## 4. Discussion

Metabolic disturbances are a critical aspect of the pathophysiology of MI and are accompanied by obesity, T2DM, dyslipidemia, and hypertension [40]. T2DM significantly increases the risk of recurrent cardiovascular events and predisposes individuals to complex MI presentation with acute left ventricular dysfunction and arrhythmias [41]. Dyslipidemia and obesity are interrelated conditions that significantly increase the risk of MI [42]. Conversely, MI worsens metabolic disruptions, creating a vicious cycle. Unhealthy dietary patterns and lifestyles are associated with cardiovascular disease, including MI, affecting the metabolic pathways [43]. Therefore, a comparison between the incidence of MI with and without metabolic disorders is needed to understand the risks associated with MI. The present study aimed to identify the genetic factors influencing MI and comorbid diseases in East Asian populations. Through the genetic correlation, colocalization, and gene expression analysis of scRNA-seq data related to MI risk, we sought to provide comprehensive insights into the genetic architecture of MI. Our findings could facilitate the development of more targeted preventive and therapeutic strategies for MI risk tailored to East Asian populations.

There is a complex interaction between genetic predisposition, lifestyle, and pharmacological treatment that influences MI risk. While our study focused on the PRS, showing the cumulative genetic impact on MI risk, we acknowledge that medication use, particularly statins for hyperlipidemia and antihypertensive agents, could modify these outcomes. We found that the PRS played a crucial role in understanding MI pathogenesis, interacting with dietary fat intake, alcohol consumption, and smoking habits. The results demonstrate how genetic predispositions influence MI susceptibility under different lifestyle habits, though these associations may be modulated by pharmacological interventions. Notably, individuals with a high PRS face increased MI risk, particularly when accompanied with low fat intake, high alcohol consumption, and current smoking. Understanding these complex interactions between genetics, lifestyle factors, and medication use enhances our knowledge of MI pathogenesis and aids in developing targeted interventions for susceptible populations. This aligns with previous studies that demonstrated that hyperglycemia and dyslipidemia are associated with PRS, interacting with low fat intake, high alcohol consumption, and current smoking [19]. However, future research should incorporate detailed medication data, especially regarding anti-dyslipidemic medication such as statins and anti-hypertensive medication, to better understand how pharmacological treatments might modify these genetic and lifestyle interactions.

In the present study, the genetic correlation analysis revealed significant associations between MI and metabolic disturbance. These findings partly clarify the complex role of genetics in MI susceptibility and its interaction with related cardiovascular conditions. Notably, specific dietary habits, such as using certain cooking oils like olive oil, have demonstrated a negative genetic correlation coefficient (r_g_ = −0.364) with MI risk, suggesting a potential protective effect against MI. Moreover, the consumption of beneficial oils rich in omega-3 and omega-6 fatty acids has been linked to various cardiovascular benefits, including anti-inflammatory, vasodilatory, anti-arrhythmic, antithrombotic, antioxidant, and anti-atherogenic effects, which could potentially lower the incidence of MI [44]. Dietary practices have been shown to influence MI susceptibility, with genetic interactions further influencing MI risk [45]. Understanding these genetic associations could help develop personalized prevention and intervention strategies for mitigating MI risk in susceptible individuals.

The colocalization analysis revealed a genetic landscape centered on rs3864814, demonstrating convergence with *STOX1* and *VPS26A*. While less established than the angiotensin-converting enzyme (*ACE*) and nitric oxide synthase (NOS) isoforms, these genes show critical links to MI pathogenesis through intricate interactions with *ACE* and *NOS*. *STOX1*, a long non-coding RNA, is pivotal in vascular remodeling due to modulating vascular smooth muscle cell (VSMC) phenotypic transformation [46]. Its interactions with *ACE* suggest potential mechanisms for regulating blood pressure and cardiac function [47,48]. *STOX1* influences VSMC plasticity through molecular pathways involving inflammatory signaling and oxidative stress responses [49], with ACE inhibition potentially preventing vascular restenosis. *VPS26A*, a retromer complex component, demonstrates significance in cardiovascular health through interactions with *NOS* isoforms (neuronal *nNOS*, inducible *iNOS*, and endothelial *eNOS*) [50,51]. Its involvement in mitochondrial dynamics and cellular energy metabolism provides insights into the mechanisms of ischemic events [52], highlighting potential roles in neuronal and cardiovascular processes.

The eQTL and colocalization analyses provide robust evidence for the functional relevance of these genetic variants. The identified rs3864814 of *KIFBP* may serve as a crucial genetic modifier of MI susceptibility, offering a mechanistic link between genetic variation and molecular dysfunction. These findings advance our understanding of MI’s molecular mechanisms by highlighting STOX1 and VPS26A as potential genetic modulators, suggesting promising avenues for future therapeutic interventions.

Previous studies have highlighted the pivotal role of cardiomyocytes, particularly those responsible for cardiac contraction, in the context of MI and ischemic injury [53]. These studies have identified key gene expression patterns that define the response of cardiomyocytes to ischemic stress, suggesting their central involvement in both the initiation of MI and the progression of injury [54]. Consistent with these findings, our analysis revealed that ventricular and atrial cardiomyocytes exhibit high disease relevance scores, confirming their significant role in the MI phenotype. The elevated expression of disease-associated genes in these cells further supports their contribution to the pathophysiology of MI, particularly in the regions of the heart responsible for contractile function. Endothelial cells, known for their critical role in maintaining vascular homeostasis [55], have also been shown to be essential players in MI, particularly in post-infarct remodeling and inflammation [56]. Our study corroborates these findings, with endothelial cells displaying high disease relevance scores, reinforcing the notion that these cells are not only integral to vascular stability, but also actively contribute to the heart’s response to ischemic damage. Post-MI, endothelial cells are involved in neovascularization and the modulation of the inflammatory response, which are crucial for the healing and repair of the infarcted myocardium. These observations shed light on the complex molecular mechanisms underlying MI and suggest potential therapeutic targets. Moreover, in alignment with previous research, our study demonstrates that the candidate gene *CHD2* exhibits significant and widespread expression across heart-related cell types [57]. The prominence of *CHD2* in neural and cardiac cells suggests that it may influence the interplay between electrical signaling and structural remodeling, both of which are vital in the progression of MI. Similarly, *RPTOR*, *DCC*, *CBX2*, *and RNF213* are key players in the disease’s molecular landscape.

## 5. Strengths and Limitations

This study offers several key strengths that enhance our understanding of MI. First, our comprehensive approach integrates genetic correlation, colocalization analysis, and cell-specific gene expression data, providing a comprehensive view of MI’s genetic basis. This integration allows a more detailed understanding of the complex interplay between genetics and cellular function in MI risk. Second, the interactions between polygenic risk scores and lifestyle factors offer valuable insights for personalized prevention strategies. Finally, gene expression patterns in specific cell types, particularly cardiomyocytes and endothelial cells, identify potential cellular targets for future therapeutic interventions. These strengths collectively contribute to advancing personalized approaches in cardiovascular medicine.

However, several limitations should be acknowledged. First, the PRS was derived from GWASs of East Asian populations. The applicability and predictive performance of this PRS in other ethnic populations might be limited due to differences in genetic architecture and LD patterns across diverse populations. Second, although the colocalization analysis revealed the potential functional significance of MI-associated variants, it relied on assumptions and annotations [58]. Incomplete or underpowered datasets could lead to underestimation or missed associations, affecting the interpretation of the results. Third, lifestyle data, especially dietary intake, were self-reported and subject to potential biases and inaccuracies. However, it is worth noting that dietary intake was measured using an SQFFQ reflecting the participants’ diet patterns and was validated with three-day dietary records [59]. Fourth, while our study included both male and female participants, the analysis did not specifically examine gender-specific differences in genetic risk and lifestyle interactions. Given the well-documented differences in MI presentation, risk factors, and outcomes between men and women, and across age groups, future studies should consider gender-stratified as well as age-based analyses to better understand how PRS and lifestyle interactions may vary by gender and age. Fifth, while medication status was identified as a factor of metabolic disturbance (T2DM, hypertension, and dyslipidemia), its potential moderating effect on genetic risk was not fully explored. Future studies should implement more nuanced approaches to assess how specific pharmacological interventions interact with genetic predispositions to influence MI risk. Furthermore, while the MI-related gene enrichment and gene set analysis of scRNA-seq datasets elucidated cellular heterogeneity and pathways [60], the limitations of scRNA-seq, such as biases and incomplete capture of myocardial complexity, should be acknowledged. Experimental validation is needed to confirm these findings.

## 6. Clinical Implications

The findings of this study have significant clinical implications for the prevention and management of MI. The PRS developed in our research offers a more precise and dynamic tool for identifying individuals at high genetic risk, enabling earlier and more targeted preventive interventions. By integrating comprehensive genetic markers and advanced statistical modeling, our PRS demonstrates enhanced predictive accuracy compared to traditional risk assessment methods. Our results on gene–environment interactions provide nuanced insights, suggesting that tailored lifestyle recommendations may be particularly beneficial for individuals with high genetic susceptibility. Specifically, we observed that targeted interventions such as moderate fat intake, controlled alcohol consumption, and smoking cessation could potentially modulate genetic risk factors. These findings underscore the critical importance of personalized prevention strategies, highlighting that individuals’ unique genetic profiles should be considered. Identifying cell-specific gene expressions, particularly in cardiomyocytes and endothelial cells, illuminates potential novel targets for pharmaceutical intervention. Genes like *STOX1* and *VPS26A* emerge as promising candidates warranting further mechanistic exploration. Our transcriptomic analysis revealed intricate regulatory networks that could potentially be manipulated to mitigate cardiovascular risk, suggesting a paradigm shift from generic to precision-based therapeutic approaches. Moreover, our comprehensive analysis of the genetic correlation between MI and metabolic syndrome-related traits provides a more holistic understanding of cardiovascular pathogenesis. By elucidating shared genetic architectures, we can develop more integrated and comprehensive prevention strategies that address underlying molecular mechanisms rather than isolated clinical manifestations.

These translational insights could significantly transform clinical practice by enabling improved patient stratification in clinical trials and more refined treatment selection. The potential to enhance both preventive and therapeutic interventions through genetic profiling represents a crucial advancement in cardiovascular medicine. Our approach not only offers a more nuanced risk assessment, but also provides a framework for developing individualized intervention protocols. The methodological innovations and biological insights presented in this study collectively suggest a transformative shift towards more personalized and precise approaches in cardiovascular medicine. By bridging genetic epidemiology, molecular biology, and clinical practice, we offer a comprehensive strategy that could substantially improve MI prevention and treatment outcomes. Critically, while our findings are promising, they also highlight the complexity of cardiovascular disease etiology and the need for continued interdisciplinary research. Future studies should focus on validating these genetic markers across diverse populations, investigating their functional implications, and developing targeted therapeutic strategies.

## 7. Conclusions

Our comprehensive study of MI risk integrates genetic, physiological, and environmental factors, revealing the complex interactions between the cardiac cells that underlie MI pathogenesis. We identified significant MI-risk genes and genetic interactions influencing disease susceptibility. Individuals with a high PRS showed adverse physiological profiles and up to threefold increased MI risk, underscoring the importance of genetic predisposition. Importantly, we found that lifestyle modifications, including a moderate intake of dietary fat rich in heart-healthy oils (>15 energy %), limited alcohol consumption, and smoking cessation, can mitigate MI risk, especially in high-PRS individuals. These findings enhance our understanding of MI determinants and offer insights into potential therapeutic targets and personalized interventions, particularly for Asian populations. Future research should validate these results across diverse ethnic groups and further explore the interplay between genetics, physiology, and environmental factors in MI risk. Our study contributes to developing more effective tailored prevention and treatment strategies for this complex cardiovascular disease.

## Figures and Tables

**Table 1:** Baseline characteristics of subjects stratified by PRS utilizing the final selection of nine optimal risk alleles: *LNX1*_rs2616417, *ELOVL2*_rs75105616, *SGCZ*_rs73201298, *KIFBP*_rs3864814, *MKRN3*_rs56730421, *CHD2*_rs201915192, *RNF213*_rs1410411669, *RPTOR*_rs7224758, and *DDC*_rs77235945.

	Control (*n* = 26,032)			Case (MI) (*n* = 1998)		
	Low PRS (*n* = 4303)	Medium PRS (*n* = 16,546)	High PRS (*n* = 4983)	Low PRS (*n* = 318)	Medium PRS (*n* = 1131)	High PRS (*n* = 549)
BMI ^a^ (kg/m^2^)	23.9 ± 2.91 ^c^	23.9 ± 2.88 ^c^	23.8 ± 2.89 ^c^	24.7 ± 2.65 ^b^	24.9 ± 2.95 ^b^	25.1 ± 3.06 ^a^*^++^
Waist circumference (cm)	80.9 ± 8.71 ^c^	80.7 ± 8.63 ^c^	80.4 ± 8.59 ^c^	84.1 ± 7.98 ^b^	84.8 ± 8.47 ^ab^	85.3 ± 8.65 ^a^**^+++^
Hip circumference (cm)	94.1 ± 5.91 ^c^	94.1 ± 5.81 ^c^	93.9 ± 5.74 ^c^	94.8 ± 5.64 ^b^	95.3 ± 5.92 ^ab^	95.6 ± 6.08 ^a^*^++^
Fasting serum glucose (mg/dL)	95.3 ± 20.5 ^c^	95.0 ± 20.3 ^c^	94.6 ± 18.5 ^c^	99.9 ± 22.7 ^b^	101 ± 26.8 ^ab^	103 ± 25.2 ^a^**^+++^
HbA1c ^b^ (%)	5.73 ± 0.74 ^c^	5.70 ± 0.73 ^c^	5.72 ± 0.74 ^c^	6.14 ± 1.04 ^b^	6.17 ± 1.08 ^b^	6.29 ± 1.15 ^a^*^+++^
Total cholesterol (mg/dL)	194 ± 38.1 ^bc^	195 ± 37.3 ^b^	195 ± 37.2 ^b^	194 ± 35.5 ^bc^	196 ± 35.4 ^b^	199 ± 35.0 ^a^***^+++^
HDL ^c^ (mg/dL)	53.8 ± 13.1 ^ab^	53.9 ± 13.2 ^a^	54.1 ± 13.2 ^a^	49.5 ± 11.9 ^b^	49.1 ± 12.0 ^bc^	48.7 ± 12.8 ^c^**^+++^
TG ^d^ (mg/dL)	120 ± 66.5 ^bc^	119 ± 65.2 ^bc^	118 ± 62.8 ^c^	123 ± 64.5 ^b^	126 ± 63.3 ^ab^	129 ± 67.1 ^a^***^+++^
SBP ^e^ (mmHg)	122 ± 15.1 ^c^	122 ± 14.9 ^c^	123 ± 14.9 ^bc^	124 ± 15.1 ^b^	125 ± 14.5 ^ab^	127 ± 15.6 ^a^**^++^
DBP ^f^ (mmHg)	75.7 ± 9.94 ^c^	75.8 ± 9.76 ^bc^	75.9 ± 9.78 ^bc^	75.8 ± 9.60 ^bc^	76.1 ± 9.62 ^b^	78.5 ± 9.21 ^a^**^+^

Values represent means ± standard deviations. PRS generated with 9 SNPs was divided into 3 categories: low (2−7), medium (8−10), and high (11−15). * Significantly different from low-PRS group at *p* < 0.05; ** *p* < 0.01; *** *p* < 0.001. ^+^ Significantly different within MI groups at *p* < 0.05; ^++^ *p* < 0.01; ^+++^ *p* < 0.001. ^a,b,c^ Means without a common letter differ in the same row by Tukey test at *p* < 0.05. ^a^ BMI, body mass index; ^b^ HbA1c, hemoglobin A1c (glycated hemoglobin); ^c^ HDL, high-density lipoprotein; ^d^ TG, triglyceride; ^e^ SBP, systolic blood pressure; ^f^ DBP, diastolic blood pressure.

**Table 2:** Logistic regression analysis of MI and its subgroups stratified by specific comorbidities using PRSs from nine-SNP model.

	Model 1			Model 2	
	Low PRS (*n* = 4712)	Medium PRS (*n* = 17,568)	High PRS (*n* = 5423)	Medium PRS (*n* = 17,568)	High PRS (*n* = 5423)
MI ^a^	1	1.597 (1.258–2.027) ***	2.765 (2.143–3.568) ***	1.716 (1.329–2.198) ***	3.074 (2.354–4.014) ***
MI + HT ^b^	1	1.331 (1.145–1.547) **	1.916 (1.621–2.264) ***	1.389 (1.189–1.624) **	2.045 (1.747–2.429) ***
MI + T2DM ^c^	1	1.488 (1.071–2.069) *	2.325 (1.628–3.324) ***	1.552 (1.107–2.176) **	2.428 (1.675–3.492) ***
MI + DL ^d^	1	1.224 (0.989–1.515)	1.767 (1.393–2.243) ***	1.282 (1.027–1.598) *	1.975 (1.499–2.447) ***
MI + OB ^e^	1	1.377 (1.194–1.636) **	2.135 (1.589–2.627) ***	1.419 (1.129–1.786) **	2.238 (1.648–2.776) ***
MI + 3GO ^f^	1	1.288 (0.775–2.162)	1.865 (1.048–2.081) *	1.295 (0.795–2.151)	1.915 (1.132–2.019) **

Values represent odd ratios and 95% confidence intervals. ^a^ Myocardial infarction. ^b^ Myocardial infarction with hypertension (HT). ^c^ Myocardial infarction with type 2 diabetes (T2DM). ^d^ Myocardial infarction with dyslipidemia (DL). ^e^ Myocardial infarction with obesity (OB). ^f^ Myocardial infarction with three or more of HT, T2DM, DL, and OB. The diagnostic criteria for hypertension were SBP≥ 140 mmHg and DBP ≥ 90 mmHg; for T2DM, fasting serum glucose ≥ 126 mg/dL; for DL, serum triglyceride, total cholesterol, or HDL concentration ≥200, ≥250, or <40 mg/dL, respectively; for OB, BMI > 25.0. PRS generated with 9 SNPs was divided into 3 categories: low (2−7), medium (8−10), and high (11−15). Low PRS was the reference for both model 1 and model 2. * Significantly different from the low-PRS group in logistic regression analysis at *p* < 0.05; ** *p* < 0.01; *** *p* < 0.001. Model 1: adjusted for age, gender, residence area, and BMI. Model 2: adjusted for age, gender, residence area, BMI, intake of energy, alcohol, and coffee, and physical activity.

**Table 3:** Adjusted MI risk and related parameters according to PRS based on optimal nine risk alleles of *LNX1*_rs2616417, *ELOVL2*_rs75105616, *SGCZ*_rs73201298, *KIFBP*_rs3864814, *MKRN3*_rs56730421, *CHD2*_rs201915192, *RNF213*_rs1410411669, *RPTOR*_rs7224758, and *DDC*_rs77235945 and interactions with the environment.

	Low PRS (*n* = 4712)	Medium PRS (*n* = 17,568)	High PRS (*n* = 5423)	PRS–Environmental Interaction *p* Value
Low energy	1	1.524 (1.188~1.954) **	2.419 (1.871~3.107) ***	0.181
High energy		2.064 (1.206~3.861) **	3.458 (1.871~4.729) ***	
Low protein	1	1.779 (1.219~2.495) **	2.857 (2.035~4.061) ***	0.816
High protein		1.442 (1.018~2.147) *	2.795 (1.902~3.989) ***	
Low CHO	1	1.545 (1.053~2.249) *	2.697 (1.789~4.062) ***	0.243
High CHO		1.662 (1.225~2.255) **	2.886 (2.077~4.018) ***	
Low fat	1	1.535 (1.103~2.137) **	2.658 (1.866~3.786) ***	0.041 *
High fat		1.661 (1.176~2.344) **	2.869 (1.987~4.145) ***	
Low natrium	1	1.726 (1.345~2.715) **	3.275 (2.258~4.756) ***	0.528
High natrium		2.062 (1.157~2.986) **	4.681 (1.036~7.645) *	
Low alcohol	1	1.775 (1.259~2.506) **	3.203 (2.229~4.609) ***	0.014 *
High alcohol		1.721 (1.165~2.558) **	2.689 (1.756~4.168) ***	
Low exercise	1	1.533 (1.078~2.159) **	2.659 (1.827~3.871) ***	0.318
High exercise		1.495 (1.051~2.479) *	2.776 (1.614~4.761) ***	
Low coffee	1	1.668 (1.185~2.811) **	3.117 (1.525~6.338) **	0.964
High coffee		1.624 (1.212~2.176) **	2.867 (2.158~3.914) ***	
Non-smoker	1	1.917 (1.183~3.562) **	3.161 (1.579~5.786) **	0.017 *
Smoker + past smoker		1.871 (1.007~3.542) *	4.069 (1.375~6.942) **	

Values represent odd ratios and 95% confidence intervals. Adjusted for age, gender, residence area, BMI, intake of energy, alcohol, and coffee, and physical activity without corresponding variables. PRS generated with 9 SNPs was divided into 3 categories: low (2−7), medium (8−10), and high (11−15). The reference was the low-PRS group. CHO, carbohydrates. Criteria for low and high definition of each parameter in interaction analysis: lower than estimated daily consumption of energy intake—less than 13% protein, 70% carbohydrate, and 15% fat; high daily energy consumption—Na intake > 2300 mg, alcohol drinking > 20 g, coffee intake > 1 cup/day, and 90 min/day moderate physical activity. * Significantly different from the major allele in logistic regression analysis at *p* < 0.05; ** *p* < 0.01; *** *p* < 0.001.

## Data Availability

The data are deposited in the Korean biobank (Osong, Korea) and were provided for the research upon request.

## Supplementary Materials

The following supporting information can be downloaded at: https://www.mdpi.com/article/10.3390/nu16244273/s1, Supplementary Table S1. Study population characteristics: stratification by MI and its comorbid subgroups. Supplementary Table S2. Lifestyles and nutrient intake of the participant groups stratified by MI and its comorbid diseases. Supplementary Table S3. Characteristics of genetic variant associated with MI risk from Korean city-based cohort GWAS. Supplementary Table S4. Characteristics of SNP-SNP interactions and their influence on MI Risk analyzed through GMDR in a Korean adult population. Supplementary Table S5. Genetic correlations between MI (n = 28,030) and other traits after Bonferroni correction.
