# Supplementary material for: Association of Metabolic Diseases and Moderate Fat Intake with Myocardial Infarction Risk
Source: Nutrients. 2024 Dec 11;16(24):4273. doi: 10.3390/nu16244273 (PMC11679910; doi:10.3390/nu16244273)
Supplement: Supplementary file 1 [file nutrients-16-04273-s001.zip › nutrients-3299738-supplementary.pdf]

Supplementary Table S1. Study population characteristics: stratification by MI and its comorbid subgroups

	Control (n=26,032)	Case (MI) (n=1,998)	MI+HT[1] (n=798)	MI+T2DM[2] (n=379)	MI+DL[3] (n=780)	MI+OB[4] (n=732)	MI+3GO[5] (n=78)	*P* value[6]
Age (years)	53.6±7.92[c]	59.9±6.83[a]	61.2±6.12[a]	61.3±6.23[a]	60.3±6.43[a]	59.8±6.52[a]	61.1±5.66[a]	<0.0001
Gender (Number, male %)	8,933(34.3)	870(52.1)	414(52.5)	237(62.5)	417(53.5)	401(54.8)	46(63.0)	<0.0001
BMI[7] (kg/m^2)	23.8±2.89[c]	24.9±2.95[bc]	25.5±2.99[b]	25.4±3.03[b]	25.2±2.88[b]	28.8±2.05[a]	27.8±2.19[a]	<0.0001
Waist circumference (cm)	80.7±8.63[c]	84.9±8.54[b]	86.5±8.36[ab]	87.0±8.62[ab]	85.9±9.07[b]	92.1±6.59[a]	92.6±6.66[a]	<0.0001
Hip circumference (cm)	94.1±5.83[c]	95.4±5.92[bc]	96.3±6.08[b]	96.1±6.25[b]	95.7±5.91[bc]	99.8±5.09[a]	99.3±5.65[a]	<0.0001
Body fat (%)	24.7±4.12[c]	27.5±3.93[b]	28.5±3.71[b]	28.3±3.88[b]	27.9±3.62[b]	31.1±2.76[a]	31.2±2.99[a]	<0.0001
Serum glucose (mg/dl)	94.9±19.9[c]	101±25.2[b]	105±27.2[b]	131±38.7[a]	102±25.4[b]	104±26.1[b]	132±40.2[a]	<0.0001
HbA1c (%)	5.71±0.73[c]	6.16±0.92[b]	6.16±0.98[b]	7.18±1.29[a]	6.14±0.89[b]	6.19±0.91[b]	7.31±1.23[a]	<0.0001
Total cholesterol (mg/dl)	193±35.3[b]	197±37.4[b]	181±37.5[bc]	173±35.7[c]	245±73.1[a]	185±38.3[b]	197±35.7[b]	<0.0001
HDL[8] (mg/dl)	53.9±13.2[a]	49.2±11.9[ab]	48.3±11.9[ab]	46.2±10.9[b]	41.3±13.5[c]	47.9±11.7[ab]	41.9±9.82[c]	0.027
TG[9](mg/dl)	118±64.9[c]	125±63.1[bc]	129±66.9[b]	131±66.6[b]	192±93.2[a]	132.7±63.7[b]	154±73.4[ab]	<0.0001
SBP[10](mmHg)	122±14.9[c]	125±14.6[b]	148±16.7[a]	126±15.4[b]	126±14.5[b]	127±14.5[b]	132±16.3[ab]	<0.0001
DBP[11](mmHg)	75.8±9.81[b]	76.1±9.46[b]	96.9±11.9[a]	76.1±9.53[b]	76.6±9.59[b]	77.8±9.17[b]	78.6±9.57[b]	<0.0001

The values represent means ± standard deviations or number of the subjects (percentage of each group).

[1]Myocardial infarction with hypertension, [2]Myocardial infarction with type 2 diabetes (T2DM), [3]Myocardial infarction with dyslipidemia, [4]Myocardial infarction with obesity. [5]Myocardial infarction with three or more of HT, T2DM, DL and OB. The diagnostic criteria for hypertension was SBP≥ 140 mmHg and DBP≥ 90 mmHg; for T2DM was fasting serum glucose≥ 126 mg/dl; for dyslipidemia was serum triglyceride, total cholesterol, or HDL concentrations were≥ 200,≥ 250, or< 40 mg/dl, respectively; for obesity was BMI> 25.0.

[6]The statistical analysis for continuous variables was conducted by one-way ANOVA after adjusting for age, sex, residence area, smoking, physical activity, intake of energy, carbohydrate percent, cholesterol, coffee, and alcohol.

[7]BMI, body mass index; [8]HDL, high-density lipoprotein; [9]TG, triglyceride; [10]SBP, systolic blood pressure; [11]DBP, diastolic blood pressure.

The statistical analysis for categorical variables was analyzed by X^2 tests.

[a,b,c] Means without a common letter differ in the same row by Tukey test at $P < 0.05$.

Supplementary Table S2. Lifestyles and nutrient intake of the participant groups stratified by MI and its comorbid diseases

	Control (n=26,032)	Case (MI) (n=1,998)	MI+HT[1] (n=798)	MI+T2DM[2] (n=379)	MI+DL[3] (n=780)	MI+OB[4] (n=732)	MI+3GO[5] (n=78)	P value[6]
Total activity (Number, %)								
None or little (<90 min/w)	11,591(44.8)	763(45.9)	356(45.9)	195(52.1)	353(46.4)	342(47.9)	35(45.2)	
Moderate (90-150 min/w)	9,560(36.9)	566(34.1)	254(32.8)	100(26.8)	241(31.7)	229(32.1)	26(32.9)	<0.0001
Heavy (>150 min/w)	4,737(18.3)	334(20.1)	165(21.3)	79(21.1)	167(21.9)	143(20.0)	17(21.9)	
Alcohol intake (g/day)								
Non-drinker (<1)	13,345(51.5)	892(53.5)	417(52.9)	189(50.1)	425(54.5)	384(52.5)	31(39.7)	
Light drinker (1-15)	969(3.74)	161(9.64)	77(9.8)	31(8.2)	88(11.3)	73(9.97)	9(12.3)	<0.0001
Moderate drinking (15-30)	6,951(26.8)	330(19.8)	157(19.9)	85(22.5)	137(17.5)	147(20.0)	21(26.5)	
Heavy drinker (>30)	4,672(18.0)	286(17.1)	137(17.4)	73(19.2)	130(16.7)	128(17.6)	17(21.5)	
Coffee intake (cups/day)								
Non-drinker (0)	4,414(17.0)	250(15.1)	105(13.4)	48(12.7)	102(13.1)	103(14.2)	9(11.1)	
Light drinker (<2)	4,743(18.3)	286(17.2)	142(18.1)	67(17.8)	147(18.9)	117(16.1)	12(15.3)	0.027
Moderate drinker (2-10)	12,443(47.9)	797(48.0)	379(48.4)	185(49.2)	372(48.1)	377(51.9)	40(52.3)	
Heavy drinker (>10)	4,367(16.8)	328(19.7)	157(20.1)	77(20.3)	154(19.9)	129(17.8)	17(21.3)	
Smoking (Number, %)								
Non-smoking	5,898(73.5)	308(62.6)	132(60.3)	69(54.8)	126(58.9)	128(59.1)	24(30.7)	
Past-smoking	1,242(15.6)	128(26.0)	58(26.5)	37(29.4)	56(26.2)	62(28.5)	36(46.2)	<0.0001
Heavy smoking	881(10.9)	56(11.4)	29(13.2)	20(15.9)	32(14.9)	27(12.4)	18(23.1)	
Energy intake (EER[7] percent)	92.4±29.2[a]	88.3±27.4[bc]	88.4±27.1[bc]	87.8±26.7[bc]	88.1±26.9[bc]	89.3±27.8[b]	85.7±28.5[c]	<0.0001
Carbohydrate intake (energy percent)	71.7±7.01[c]	74.1±6.72[a]	73.3±6.65[b]	72.7±7.29[bc]	73.4±6.65[b]	72.3±7.19[bc]	72.2±7.76[bc]	<0.0001

Protein (energy percent)	13.9±2.82[a]	13.2±2.64[b]	13.2±2.64[b]	13.2±2.77[b]	13.1±2.50[b]	13.3±5.52[b]	13.4±3.04[b]	0.003
Fat intake (energy percent)	14.4±5.42[a]	12.8±5.23[bc]	12.5±5.07[bc]	12.8±5.48[bc]	12.2±5.17[c]	13.2±5.52[b]	13.1±5.92[b]	<0.0001
Fiber(g/1000 kcal)	5.69±2.77[a]	5.61±2.82[a]	5.60±2.94[a]	5.33±2.59[b]	5.58±2.56[a]	5.64±2.73[a]	4.81±1.86[c]	<0.0001
Na(mg/1000 kcal)	2,431±1,367[a]	2,369±1,410[ab]	2,359±1,396[ab]	2,253±1,242[b]	2,358±1,317[ab]	2,458±1,433[a]	2,151±1,097[c]	0.048
Ca(mg/day)	442±252[a]	418±236[b]	415±251[b]	396±222[c]	410±214[bc]	419±225[b]	371±192[d]	<0.0001
P(mg/day)	887±346[a]	845±321[bc]	843±335[bc]	831±321[c]	837±306[bc]	858±316[b]	811±325[d]	<0.0001
Fe(mg/day)	9.94±4.76[a]	9.58±4.56[b]	9.54±4.77[b]	9.38±4.61[c]	9.52±4.31[b]	9.76±4.45[ab]	8.88±4.99[d]	0.004
K(mg/day)	2,222±1,016[a]	2,135±982[b]	2,133±1,021[b]	2,064±933[c]	2,117±904[bc]	2,163±912[ab]	1,944±774[d]	0.019

The values represent means ± standard deviations or number of the subjects (percentage of each group).
[1]Myocardial infarction with hypertension, [2]Myocardial infarction with type 2 diabetes (T2DM), [3]Myocardial infarction with dyslipidemia, [4]Myocardial infarction with obesity. [5]Myocardial infarction with three or more of HT, T2DM, DL and OB. The diagnostic criteria for hypertension was SBP≥ 140 mmHg and DBP≥ 90 mmHg; for T2DM was fasting serum glucose≥ 126 mg/dl; for dyslipidemia was serum triglyceride, total cholesterol, or HDL concentrations were≥ 200,≥ 250, or< 40 mg/dl, respectively; for obesity was BMI> 25.0.
[6]The statistical analysis for continuous variables was conducted by one-way ANOVA after adjusting for age, sex, residence area, smoking, physical activity, intake of energy, carbohydrate percent, cholesterol, coffee, and alcohol.
[7]EER, estimated energy requirement.
The statistical analysis for categorical variables was analyzed by X^2 tests.
[a,b,c] Means without a common letter differ in the same row by Tukey test at *P*< 0.05.

Supplementary Table S3. Characteristics of genetic variant associated with MI risk from Korean city-based cohort GWAS

CHR[a]	SNP[b]	Position	Minor[c]	Major[d]	OR[e]	*P*_adjust[f]	MAF[g]	HWE_*P*[h]	Gene	Functional Consequence
4	rs2616417	54488165	A	G	0.768	7.61E-06	0.173	0.707	*LNX1*[1]	intron
6	rs75105616	10992286	G	A	2.008	5.86E-08	0.018	0.403	*ELOVL2*[2]	intron
8	rs73201298	14789272	T	C	1.211	6.49E-06	0.343	0.231	*SGCZ*[3]	intron
10	rs3864814	70778486	A	C	0.563	3.13E-08	0.086	0.773	*KIFBP*[4]	imd transcript
15	rs56730421	23875145	C	T	1.304	9.86E-07	0.165	0.402	*MKRN3*[5]	3 prime utr
15	rs201915192	93520839	C	T	0.699	6.64E-08	0.181	0.243	*CHD2*[6]	nmd transcript
17	rs74608211	77760583	T	C	2.321	1.24E-08	0.011	0.127	*CBX2*[7]	3 prime utr
17	rs1410411669	78342233	T	G	0.818	9.64E-06	0.312	0.208	*RNF213*[8]	non coding transcript exon
17	rs7224758	78761732	A	G	0.735	8.03E-06	0.124	0.581	*RPTOR*[9]	nmd transcript
18	rs77235945	50695373	C	T	1.588	2.10E-07	0.046	0.744	*DDC*[10]	nmd transcript

MI,myocardial infarction; [a]Chromosome; [b]Single nucleotide polymorphism; [c]Minor allele of haplotype; [d]Major allele of haplotype; [e]Odds ratio; [f]Statistical significance of MI after adjusting for age, gender, residence area and activity; [g]Minor allele frequency; [h]Hardy-Weinberg equilibrium. [1]*LNX1*, ligand of numb-protein X 1; [2]*ELOVL2*, ELOVL fatty acid elongase 2; [3]*SGCZ*, sarcoglycan zeta; [4]*KIFBP*, kinesin family binding protein; [5]*MKRN3*, makorin ring finger protein 3; [6]*CHD2,* chromodomain helicase DNA binding protein 2*;* [7]*CBX2,* chromobox 2*;* [8]*RNF213*, ring finger protein 213; [9]*RPTOR,* regulatory associated protein of MTOR complex 1*;* [10]*DDC, Dopa Decarboxylase*

Supplementary Table S4. Characteristics of SNP-SNP interactions and their influence on MI Risk analyzed through GMDR in a Korean adult population

GMDR	Adjusted for sex, age, residential area				Adjusted for sex, age, residential area, activity, education, smoking, EER[a], alcohol			
Model	TRBA[b]	TEBA[c]	*P* value[1]	CVC[d]	TRBA	TEBA	*P* value[2]	CVC
*CHD2*_rs201915192	0.5283	0.5176	10 (0.0010)	10/10	0.5276	0.4976	10 (0.0010)	10/10
Model 1 plus *LNX1*_rs2616417	0.5334	0.5187	10 (0.0010)	7/10	0.5219	0.5123	10 (0.0010)	7/10
Model 1 plus *MKRN3*_rs56730421 *RNF213*_rs1410411669	0.5412	0.5140	10 (0.0010)	6/10	0.5328	0.5135	10 (0.0010)	6/10
Model 1 plus *SGCZ*_rs73201298 *RNF213*_rs1410411669 *RPTOR*_rs7224758	0.5495	0.5129	9 (0.0107)	6/10	0.5254	0.5117	9 (0.0107)	6/10
Model 2 plus Model 4	0.5648	0.5110	8 (0.0547)	7/10	0.5583	0.5024	8 (0.0547)	7/10
Model 3 plus Model 5	0.5849	0.5091	7 (0.1719)	10/10	0.5763	0.5034	7 (0.1719)	10/10
Model 6 plus *KIFBP*_rs3864814	0.6457	0.5222	9 (0.0107)	10/10	0.6362	0.5165	9 (0.0107)	10/10
Model 7 plus *DCC*_rs77235945	0.6824	0.5119	9 (0.0107)	10/10	0.6764	0.5105	9 (0.0107)	10/10
Model 8 plus *ELOVL2*_rs75105616	0.7332	0.5053	10 (0.0010)	10/10	0.8674	0.5021	10 (0.0010)	10/10
Model 9 plus *CBX2*_rs74608211	0.7412	0.5066	9 (0.0107)	10/10	0.7815	0.5045	9 (0.0107)	10/10

SNP, single-nucleotide polymorphism; MI,myocardial infarction; [a]EER, estimated energy requirement; [b]TEBA, test balance accuracy; [c]TRBA, trained balanced accuracy; [d]CVC, cross-validation consistency.

[1]TEBA by Chisqaure test in model 1. [2] TEBA by Chisquare test in model 2.

LNX1, ligand of numb-protein X 1; *ELOVL2*, ELOVL fatty acid elongase 2; *SGCZ*, sarcoglycan zeta; *KIFBP*, kinesin family binding protein; *MKRN3*, makorin ring finger protein 3; *CHD2*, chromodomain helicase DNA binding protein 2; *CBX2*, chromobox 2; *RNF213*, ring finger protein 213; *RPTOR*, regulatory associated protein of MTOR complex 1; *DDC,* Dopa Decarboxylase

Supplementary Table S5. Genetic correlations between MI (n = 28,030) and other traits after Bonferroni correction

Trait	Sample size	Cohort	Category	Myocardial infarction (LDSC)				
				r_g	s.e.	z-score	*P*	FDR adjusted *P*
Coronary artery disease	212,453	BioBank Japan	Health indicator	0.759	0.123	24.3	3.55E-130	3.08E-128
Peripheral artery disease	212,453	BioBank Japan	Health indicator	0.614	0.076	11.9	1.15E-32	5.01E-31
Atrial Fibrillation	36,792	BioBank Japan	Health indicator	0.605	0.068	15.1	1.52E-51	4.41E-50
Hyper-LDL cholesterolemia	72,132	National Biobank of Korea	Health indicator	0.537	0.149	10.9	8.47E-28	1.87E-26
Arrhythmia	212,453	BioBank Japan	Health indicator	0.464	0.079	5.87	4.34E-09	7.53E-08
Hypertension	57,395	National Biobank of Korea	Health indicator	0.428	0.137	11.5	1.72E-30	2.49E-29
Current smoking	15,203	National Biobank of Korea	Life style	0.407	0.054	4.84	1.59E-06	1.97E-05
Type 2 diabetes	67,127	National Biobank of Korea	Health indicator	0.386	0.049	7.86	6.43E-15	6.95E-14
Dyslipidemia	56,348	National Biobank of Korea	Health indicator	0.349	0.189	3.92	8.96E-05	8.68E-04
Past smoker	15,993	National Biobank of Korea	Life style	0.309	0.065	3.63	2.84E-04	0.002
Alcohol drinker	72,151	National Biobank of Korea	Diets	0.290	0.034	4.55	5.47E-07	4.32E-05
Body mass index (BMI)	72,282	National Biobank of Korea	Health indicator	0.282	0.077	7.59	3.09E-14	2.24E-13
Body fat percentage	72,222	National Biobank of Korea	Health indicator	0.282	0.025	7.91	2.53E-15	1.66E-14
Recent feelings of tiredness or low energy	56,324	National Biobank of Korea	Life style	0.281	0.021	4.64	3.42E-07	2.12E-05
Variation in diet	80,263	National Biobank of Korea	Diets	0.279	0.025	5.05	4.51E-07	2.60E-06
Feeling disturbed or anxious at night	32,544	National Biobank of Korea	Life style	0.271	0.019	3.93	8.47E-05	4.60E-04
Sleeplessness	58,264	National Biobank of Korea	Life style	0.222	0.015	4.82	1.42E-07	7.25E-06

Feeling unhappy and depressed	42,706	National Biobank of Korea	Life style	0.127	0.011	2.05	0.044	0.009
Sensitivity or hurt feelings	51,653	National Biobank of Korea	Life style	0.126	0.009	2.55	0.011	0.049
Vitamin and mineral supplements	11,960	National Biobank of Korea	Diets	-0.133	0.034	-2.41	0.016	0.032
Regular activity	84,243	National Biobank of Korea	Life style	-0.194	0.067	-3.92	9.64E-05	3.94E-04
Feeling comfortable and healthy	80,128	National Biobank of Korea	Life style	-0.216	0.101	-3.21	0.001	0.005
Tea intake	78,429	National Biobank of Korea	Diets	-0.227	0.095	-3.01	0.002	0.009
Coffee intake	80,285	National Biobank of Korea	Diets	-0.248	0.087	-4.18	2.95E-05	1.06E-04
Drinking milk more than three times a week	40,902	National Biobank of Korea	Diets	-0.296	0.126	-4.56	5.09E-07	1.77E-05
Eating peanuts, almonds, pine nuts	58,327	National Biobank of Korea	Diets	-0.311	0.083	-4.83	1.39E-07	4.65E-06
Eating eggs/quail eggs more than three times a week	21,360	National Biobank of Korea	Diets	-0.324	0.074	-5.09	3.53E-07	1.12E-06
Using olive oil, sesame oil, perilla oil for cooking	13,790	National Biobank of Korea	Diets	-0.364	0.077	-4.78	1.78E-07	5.53E-06
Never smoked	17,905	National Biobank of Korea	Life style	-0.3825	0.073	-3.73	1.92E-05	5.76E-04
No history of myocardial infarction	58,410	National Biobank of Korea	Health indicator	-0.51	0.103	-14.3	1.55E-44	4.48E-44

LDSC, linkage disequilibrium score regression; r_g, genetic correlation coefficients; s.e., standard error; *P*, *P*-value of a two-sided test after Bonferroni correction; FDR, false discovery rate.
